# Towards a comprehensive estimate of national spending on prevention

**DOI:** 10.1186/1471-2458-7-252

**Published:** 2007-09-20

**Authors:** Esther W de Bekker-Grob, Johan J Polder, Johan P Mackenbach, Willem Jan Meerding

**Affiliations:** 1 Department of Public Health, Erasmus MC, Rotterdam, the Netherlands; 2 National Institute for Public Health and the Environment, Bilthoven, the Netherlands; 3 Department Tranzo, Tilburg University, Tilburg, the Netherlands

## Abstract

**Background:**

Comprehensive information about national spending on prevention is crucial for health policy development and evaluation. This study provides a comprehensive overview of prevention spending in the Netherlands, including those activities beyond the national health accounts.

**Methods:**

National spending on health-related primary and secondary preventive activities was examined by funding source with the use of national statistics, government reports, sector reports, and data from individual health associations and corporations, public services, occupational health services, and personal prevention. Costs were broken down by diseases, age groups and gender using population-attributable risks and other key variables.

**Results:**

Total expenditures on prevention were €12.5 billion or €769 per capita in the Netherlands in 2003, of which 20% was included in the national health accounts. 82% was spent on health protection, 16% on disease prevention, and 2% on health promotion activities. Most of the spending was aimed at the prevention of infectious diseases (34%) and acute physical injuries (29%). Per capita spending on prevention increased steeply by age.

**Conclusion:**

Total expenditure on health-related prevention is much higher than normally reported due to the inclusion of health protection activities beyond the national health accounts. The allocative efficiency of prevention spending, particularly the high costs of health protection and the low costs of health promotion activities, should be addressed with information on their relative cost effectiveness.

## Background

In the 20^th ^century, preventive activities resulted in major improvements in public health [1]. Among the most significant activities are sewerage systems, road safety, vaccinations and clean water technologies. Particularly the latter have contributed considerably to the decrease in mortality in the late 19^th ^and early 20^th ^century [2].

In contrast to their public health impact, the share of prevention in total health expenditures is often claimed to be small [3]. In the USA, expenditures on prevention included in the national health accounts (NHA) were estimated at 3% of total national health expenditure in 1988 [4]. This share was recently estimated for 22 OECD countries, ranging from 0.6% (Italy) to 8% (Canada) [5]. However, expenditures that include only prevention within the NHA are an underestimate as major public health interventions (such as clean water and air, sanitation, and road traffic safety) are managed by different departments outside the health care sector. This was illustrated by the CDC report mentioned above, in which the total amount spent on prevention was almost double the prevention expenditures within the NHA [4].

New preventive and curative health technologies are being developed continuously, and health authorities are faced with decisions about allocating resources towards these technologies, in addition to (or as substitute for) existing interventions. This requires knowledge on the effectiveness, resource use, and costs of new and existing technologies. Differences in cost effectiveness indicate that we could save lives more effectively and efficiently [6]. Regarding prevention, the search for efficiency (costs per unit of health gained) is complicated because it extends to other policy budgets, and the health claims of prevention have to compete with other policy objectives.

Accordingly, comprehensive information about national spending on prevention is crucial for health policy development and evaluation. We present here an overview of the costs of health-related preventive activities in the Netherlands in 2003, both inside and outside the health care sector, as a framework for more detailed (cost-effectiveness) analyses. Costs are broken down by different categories, disease groups, age and gender, and will also be discussed from the perspective of expenditure on curative services and long-term care.

## Methods

### Identification of preventive activities

We considered preventive activities conducted by (semi-)government, trade and industry, and consumers (societal perspective). We identified these preventive activities as follows. First, we consulted overview articles or (policy) reports [7-12], that had the explicit aim of giving an integral description of preventive health activities. Supplementary, we consulted experts to fill up the overview of different preventive activities in the Netherlands as good as possible. These experts were policy makers of different Ministries, scientific researchers (e.g. in the field of public health, environment, or medicine), and employees of different institutes (e.g. fire department, police, or center for alcohol and drugs dependence). Finally, we searched on the Internet for more detail on the preventive activities suggested by these experts. We used the database Medline and the search machine Google to identify published studies or (policy) reports about these activities.

We included primary and secondary preventive activities (except for preventive medication, such as cholesterol suppressants and anti-hypertensives, for which we included primary prevention only). Tertiary preventive activities were excluded because these are difficult to separate from usual patient care.

Preventive activities performed by actors integrated in the NHA (e.g. dental practices, mental health care, GP practices, and Occupational Health Services) were all seen as relevant and included. Preventive activities considered outside the NHA (e.g. rule enforcement of drugs, alcohol, and smoking, activities to guarantee traffic safety, and reduction of noise pollution) all aim to protect or promote health. However, some interventions are not exclusively aimed at promoting health, but also serve other sources of welfare, such as a clean (e.g. domestic waste disposal), comfortable (e.g. noise barriers), and safe (e.g. prevention of violence) environment, either physical or social. We included those interventions for which the promotion of health is the principal policy objective or has been the dominant motive by the time of introduction, even though these interventions were not exclusively aimed at promoting health. For this, we relied to a great extent on expert opinions. Although the existence of multiple policy objectives is evident for specific interventions, valid criteria are lacking to break down the expenditures of these interventions by separate policy objectives.

Preventive activities aimed at sports were partly included (e.g. promoting sport as a public initiative) due to a dominant public health aim, whereas this was not the case for sports in general (e.g. health clubs, club membership, time investment at sports, and expenditures on sport material). Education was not labeled as prevention, although schools can play an important role in promoting healthy lifestyles regarding nutrition and sexual behavior.

Although excise duties (such as tobacco and alcohol taxes) are important public health instruments, they were not included since from a societal perspective they must be regarded as transfer payments rather than real (opportunity) costs.

### Data collection

Various sources were used to quantify the expenditures on selected preventive measures: national statistics [10], government reports [13-15], sector reports [8,16], and data from individual health associations and corporations [17-27], public agencies [28,29], occupational health services [30,31], and personal prevention. An overview of preventive activities, data sources, and types of cost calculation method is given in Additional file 1. All expenditures relate to operational expenditures and include personnel, material, equipment, housing, maintenance and overhead costs. All data are from the year 2003. First, we collected existing financial data of preventive activities on the national level to avoid double counting. We used annual reports (e.g. Benchmark report Municipal Health Services [29], Netherlands Environmental Health Agency [8], Dutch anti-smoking group STIVORO [26], Netherlands Nutrition Center[24]) and also consulted experts (Dutch Society for Oral Health Education, Ministry of Education, Culture and Science, Food and Consumer Product Safety Authority). Second, if no data were available at national level, we collected financial data on preventive activities at regional level. We used annual reports and (official) balance sheets of different providers or collected financial data by consulting experts (e.g. Centers for Alcohol and Drugs dependence, fire departments). If annual reports or financial data were not available for all providers performing a certain preventive activity (e.g. not all Centers for Alcohol and Drugs dependence responded), we extrapolated the expenditures by using the related population figures. Finally, expenditures on personal preventive activities were collected with the help of retail data. We asked providers, or used statistics, concerning how much of a specific product (e.g. cholesterol suppressants, condoms, vitamins) was sold.

### Data analysis

On aggregate level we analyzed the distribution of preventive expenditure from a societal perspective as well as from a more restricted health care perspective as used in NHA and in the System of Health Accounts (SHA) developed by the OECD. Then, all identified preventive measures were classified as either health promotion, health protection, or disease prevention. Health promotion is aimed at promoting healthy behavior through information and education; health protection is aimed at reducing exposure to environmental health risks by legislation, control, and interventions; disease prevention includes preventive medication, vaccination, and screening. Health promotion and health protection are both aimed at a specific determinant and not directly at a specific disease. Disease prevention is disease specific, and includes primary preventive activities (i.e. aimed at a determinant of a specific disease) as well as secondary preventive activities (i.e. aimed at a specific disease). Although most preventive activities could be uniquely classified (e.g. information about healthy food, smoking cessation campaigns, and swimming education could be classified entirely to health promotion; rule enforcements of employment conditions, drugs, alcohol and smoking could be classified to health protection; National Vaccine Program, cervical cancer screening program, and blood pressure suppressants could all be classified to disease prevention), some preventive measures overlapped multiple categories. For instance, expenditure on youth health care by municipal health services was divided into disease prevention (screening activities, 85%) and health promotion (15%) based on contact guidelines and expert consultation. Preventive activities of occupational health services were entirely allocated to health protection, although a minority of activities can be regarded as disease prevention (e.g. health check-ups) or health promotion (e.g. education about work posture and physical load); this was guided by expert opinion since registration data were unavailable. Similarly, preventive activities by fire departments were classified as health protection because they are predominantly directed as risk assessment and enforcement of fire safety regulations, whereas no specific information was available on information and education activities undertaken by fire brigades.

Preventive expenditures were allocated to age groups (0–19, 20–44, 45–64, 65+ years), gender, and disease groups using key variables. For example, we used figures on screening participation to allocate expenditures on the national breast cancer screening program to women in the different age groups. Similarly, expenditures of cholesterol-lowering therapy were allocated to age and gender by drugs-dispensing data from the 2003 national registry on pharmaceutical care [32]. Prenatal screening expenditures have been allocated to pregnant women instead of the (unborn) child. Expenditures on health protection were allocated to age and gender by using population figures and, more specifically, by the numbers of employees in the case of health protection at the work place.

Disease groups were in line with the cost-of-illness approach defined according to the 17 chapters of the ICD-9 (International statistical Classification of Diseases, injuries and causes of death)[33].

Most preventive measures could uniquely be related to disease groups (e.g. all vaccinations, screening for TBC, and sexually-transmitted diseases could be classified to 'infectious diseases'; preventive measures related to traffic safety, sport injuries and fire safety could be classified to 'injuries'). Preventive measures aimed at addiction and gambling dependence were consistently allocated to mental disorders.

Some other preventive measures, however, relate to risk factors for multiple diseases, such as smoking and obesity. The costs of these preventive measures were allocated to each disease group using the population attributable risk (PAR) for mortality, which is the proportion of cause-specific deaths that is attributable to a risk factor (Additional file 2 presents an example of allocating costs of anti-smoking medication to different disease groups). According to this method the prevention costs that belong to one disease group (cause of death) are equal to the prevention costs related to a risk factor multiplied by the relative number of cause-specific deaths within the total number of deaths attributable to the risk factor. This analysis was done according to age and gender. PARs were calculated with relative risks and prevalence of risk factors from the literature, as used for the national Public Health Forecasts [34]. In general, the formula for the PAR is:

PAR=P(E)∗(RR−1)1+P(E)∗(RR−1)
 MathType@MTEF@5@5@+=feaafiart1ev1aaatCvAUfKttLearuWrP9MDH5MBPbIqV92AaeXatLxBI9gBaebbnrfifHhDYfgasaacH8akY=wiFfYdH8Gipec8Eeeu0xXdbba9frFj0=OqFfea0dXdd9vqai=hGuQ8kuc9pgc9s8qqaq=dirpe0xb9q8qiLsFr0=vr0=vr0dc8meaabaqaciaacaGaaeqabaqabeGadaaakeaacqqGqbaucqqGbbqqcqqGsbGucqGH9aqpdaWcaaqaaiabbcfaqjabcIcaOiabbweafjabcMcaPiabgEHiQiabcIcaOiabbkfasjabbkfasjabgkHiTiabigdaXiabcMcaPaqaaiabigdaXiabgUcaRiabbcfaqjabcIcaOiabbweafjabcMcaPiabgEHiQiabcIcaOiabbkfasjabbkfasjabgkHiTiabigdaXiabcMcaPaaaaaa@486B@

where P(E) is the proportion of the general population exposed to a particular agent, and the RR is the relative risk (or rate ratio, or odds ratio (OR)) of disease or death for the exposed versus nonexposed. For more information, see also the study of Steenland et al. [35] who estimated the annual deaths associated with occupation by calculation of PARs.

Not all preventive activities could be allocated to relevant disease groups mainly because available data lacked sufficient detail, e.g. use of vitamins, general medical examinations, youth health care (excluding vaccination), housing quality inspectorate, general health promotion, and occupational prevention. These were classified as "not disease specific".

## Results

In the Netherlands, total expenditures on prevention were estimated at €12.5 billion in 2003, equal to €769 per inhabitant capita (Table 1). Of these, €628 per capita (82%) was spent on health protection, €126 per capita (16%) on disease prevention, and €15 per capita (2%) on health promotion. The amounts spent on health protection were mainly aimed at environmental safety (€120.5 per capita; e.g. reduction of soil pollution due to agricultural and industrial activities (including management of chemicals, heavy metals, and ammonia)), traffic safety (€99.7 per capita), domestic waste disposal (€99.1 per capita), and the quality of air (€98.9; e.g. reduction of air pollution by industry and traffic). Among disease prevention activities, the highest amounts were spent on cardiovascular disease control (predominantly statins and antihypertensives), and preventive dental health. The amounts spent on health promotion were mainly aimed at prevention of mental disorders (including prevention of suicide, and supporting children of parents with mental problems) and promoting non-specific general health. Often more than one preventive method was used to control a risk factor or disease. For instance, traffic safety is stimulated by health protection measures (airbags, speed limits, and so on: €99.7 per capita), and by health promotion (€0.2 per capita).

**Table 1:** National spending on prevention by method, in the Netherlands in 2003, in € per capita

*Interventions*	*Health promotion (€/capita)*	*Health protection (€/capita)*	*Disease prevention (€/capita)*	*Total (€/capita)*	*Total (%)*
***Risk factor***					
Smoking cessation	0.99	0.10	0.23		
Alcohol dependence	0.50	0.10		0.61	0.08
Drugs dependence	0.31	0.10		0.42	0.05
Gambling dependence	0.11			0.11	0.01
Healthy food promotion and overweight	0.75			0.75	0.10
Physical activity and sport	1.49			1.49	0.19
Youth health care	1.55		8.76	10.31	1.34
Preventive dental health	0.01		30.17	30.18	3.92
Sexual health, contraception <21 years	1.19		0.73	1.91	0.25
Traffic safety	0.19	99.69		99.87	12.99
Fire prevention	0.19	3.52		3.71	0.48
Prevention of sport injuries	0.41			0.41	0.05
Prevention of home and leisure injuries	0.36	0.45		0.81	0.11
Occupational health		14.99		14.99	1.95
Violence		1.28		1.28	0.17
Noise pollution		34.48		34.48	4.48
Drinking water system and swimming water		91.63		91.63	11.91
Domestic waste disposal		99.06		99.06	12.88
Sewerage system		49.22		49.22	6.40
Food safety		9.63		9.63	1.25
Air quality		98.89		98.89	12.86
Housing quality		4.56		4.56	0.59
Environmental safety		120.50		120.50	15.67
General health (non-specific)	2.19		5.78	7.97	1.04
***Disease***					
Mental disorders	3.73			3.73	0.48
Cancer prevention	0.95		4.26	5.21	0.68
Diabetes prevention	0.05			0.05	0.01
Cardiovascular disease control (statins,			55.70	55.70	7.24
antihypertensives)					
Congenital and perinatal conditions, and			7.12	7.12	0.93
pregnancy complications					
Osteoporosis			4.19	4.19	0.54
Infectious diseases: vaccinations and			8.96	8.96	1.17
screening					
**Total (€/capita)**	**14.97**	**628.20**	**125.90**	**769.08**	**100.00**

Of all expenditure on prevention, 20% (€152 per capita) was made within the health care system and accounted for 4.3% of total health expenditure. This part of preventive expenditure was dominated by disease-prevention activities such as cholesterol-lowering therapy, antihypertensive treatment, cancer screening, and vaccination (82%). A much smaller part was spent on health protection activities (10%, environmental safety and control by Municipal Health Services and activities by occupational health services) and on health promotion (8%).

From a societal perspective expenditure on prevention exceeds the investments on prevention undertaken by health providers within the scope of the health care system. Table 2 shows the difference between our societal estimates and the amounts that were labeled as prevention in the SHA. According to the definitions of the SHA, total spending on prevention was €139 per inhabitant. However, according to our approach total spending on prevention was €769 per capita. Especially inclusion of health protection measures outside the health care system and inclusion of preventive medication caused this difference.

**Table 2:** Estimation of national spending on prevention in case of the SHA approach and the present study approach, in the Netherlands in 2003, in € per capita

	Providers	SHA (€/capita)	Study (€/capita)
HP.1	Hospitals		
	Mental health and substance abuse hospitals	0.68	2.33
HP.2	Nursing and residential care facilities	0.00	0.00
HP.3	Providers of ambulatory health care		
	Offices of physicians	7.53	7.76
	Offices of dentists	22.97	30.07
	Providers of home care services	18.53	3.52
	All other providers of ambulatory care	0.68	7.58
HP.4	Retail sale and other providers of medical goods	0.00	60.59
HP.5	Provision and administration of public health programs		
	Municipal Health Services	37.06	17.67
	Insitutions for cervical cancer research	1.30	1.31
	Insitutions for breast cancer research	2.41	2.59
HP.6	General health administration and insurance	0.00	0.00
HP.7	Other industries (rest of the economy)		
	Occupational health care services	46.69	13.40
	All other industries as secondary producers of health care	1.24	0.00
HP.9	Rest of the world	0.00	0.00
HP.10	Providers beyond SHA		
	Health promotion organisation/association		4.76
	Authorities (e.g. Ministries, police, municipalities)		265.62
	Traffic and transport sector		47.04
	Building sector		0.18
	Energy sector		11.67
	Trade and service sector		1.53
	Industry sector		30.96
	Agriculture sector		7.13
	Environmental service sector		98.94
	Refineries		4.48
	Drinking-water companies		90.42
	Consumers		59.47
	**Total**	**139.08**	**769.00**

**Figure 1:**
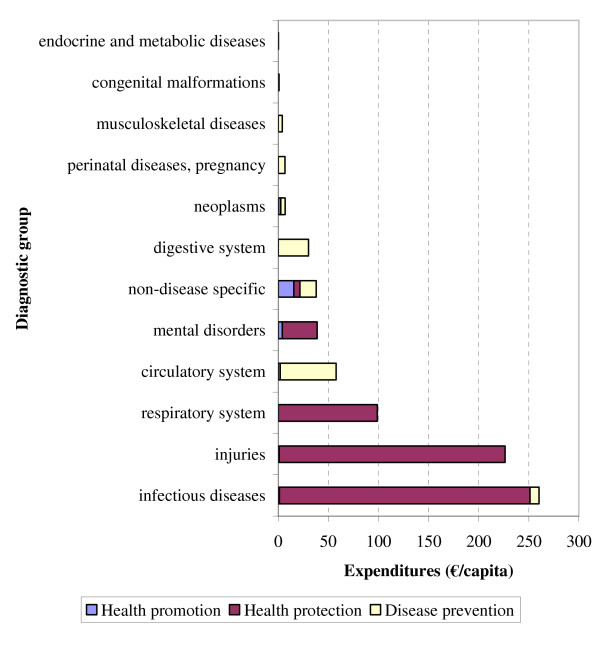
National spending on preventive methods by disease group (ICD-9 chapters), in the Netherlands in 2003, in € per capita.

Most of the spending on preventive measures was aimed at prevention of infectious diseases (34%), followed by injury (29%), respiratory diseases (13%), cardiovascular diseases (8%) and mental disorders (5%) (Figure 1).

**Figure 2:**
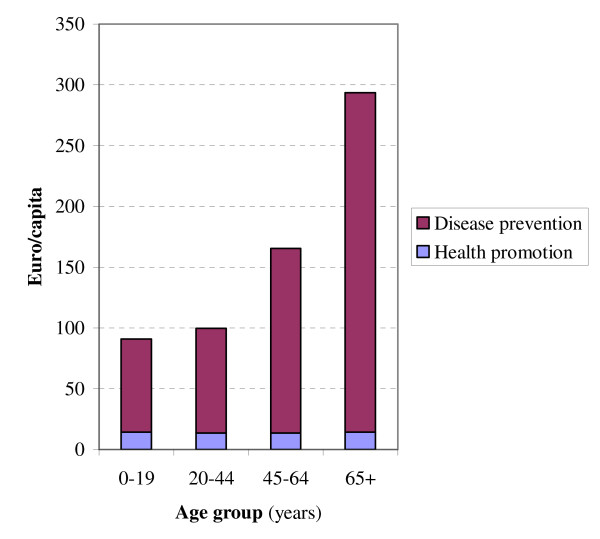
National spending on health promotion and disease prevention by age group, in the Netherlands in 2003, in € per capita.

Preventive expenditures spent on health protection measures were similar across all age groups, because these measures are aimed at the general population. The amounts spent per person on health promotion measures are slightly higher for children and older persons than for younger adults (Figure 2). This reflects, for example, swimming education for schoolchildren and prevention of home and leisure injuries in older people. Despite that many screening activities and vaccination programs are directed at children, spending on disease prevention is substantially lower among children than among those aged 45 years and over. This is because of the high expenditures associated with the use of preventive medication. Amounts spent on prevention were slightly higher for women, mainly due to screening for breast cancer and cervical cancer, pregnancy monitoring, and the use of contraceptives by teenagers (females <21 years). Females, compared with males, also used more antihypertensives, vitamins and osteoporosis medication.

**Figure 3:**
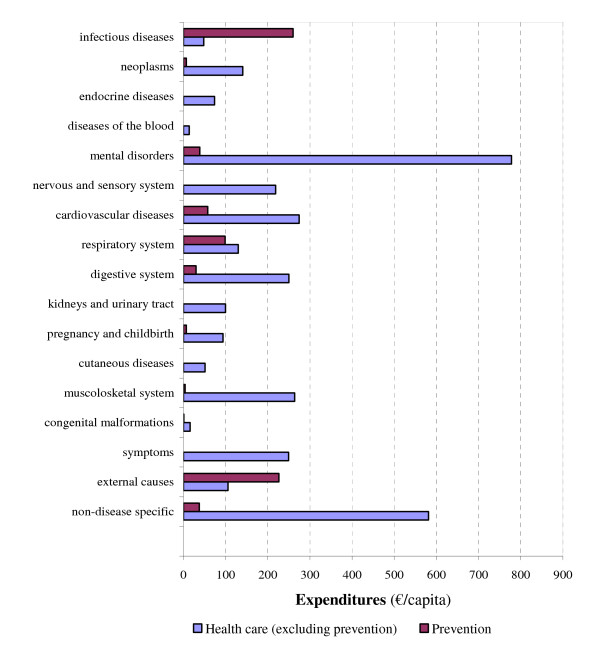
National spending on prevention versus curative services and care by disease group (ICD-9 chapters), in the Netherlands in 2003, in € per capita.

Figure 3 shows the national spending on prevention compared with expenditures on curative services and long-term care, by disease group. Only for infectious diseases and for acute physical injuries did the national spending on prevention exceed the health expenditure of the particular disease groups. These high preventive expenditures reflect investments in expensive but effective measures such as domestic waste disposal, clean water technology, and traffic safety. Expenditure on cure and care for mental disorders and diseases of the musculoskeletal system was much higher compared with prevention costs.

## Discussion

In the Netherlands expenditure on prevention in 2003 amounted to €769 per inhabitant (€12.5 billion in total), which is much higher than usually reported. This is due to the inclusion of health protection activities beyond the NHA (e.g. health protection interventions related to traffic safety, drinking water system, and air quality), that are fourfold the prevention expenditures within the NHA (4.3% of all health care costs). Most expenditures of preventive measures were aimed at infectious diseases (34%; e.g. domestic waste disposal, clean water technologies, and vaccinations) and acute physical injuries (29%; e.g. environmental safety and traffic safety). The expenditures on disease prevention were lowest in children and adolescents, and increased steeply with age. Amounts spent on prevention were slightly higher for women. Only for infectious diseases and injuries were expenditures on prevention higher than expenditures on curative services and care services.

Although we aimed for a complete overview of spending on preventive measures, data were unavailable for specific police expenditures (e.g. maintenance of environmental and traffic regulation, prevention of violence), prevention by general practitioners (e.g. blood pressure surveillance, health education), and industrial spending on food safety. Data for specific consumer expenditures were also lacking due to (many) different providers supplying one specific preventive product in combination with no access to the financial data concerning the product. Therefore, it was not possible to collect (reliable) financial data on preventive products such as fire alarms, sunscreen, car safety seats, prescription-free vitamins, and bicycle helmets. Expenditures on sports products (e.g. shoes, skis) were ignored, because in this study these products were not labelled as preventive activities. As a result, our figures are still an underestimate. On the other hand, we included interventions that were not exclusively aimed at promoting health, but also serve other policy objectives (e.g. promotion of physical activity was also aimed at promoting social developments, and the sewerage system was also aimed at promoting a clean environment). The rationale for their inclusion was that, in these instances, health objectives are considered to dominate other policy objectives, and valid criteria are lacking to break down the expenditures of these interventions by separate policy objectives. If interventions directed at multiple policy objectives would be excluded, the total prevention expenditures would then be €160 per capita (€2.6 billion for the Dutch society) instead of €769 per capita (€12.5 billion for the Dutch society). Although the inclusion of health protection measures beyond the NHA is questionable, in our opinion the inclusion of these measures is justified based on their positive contribution to public health [36,37].

A second limitation of our study is that we assumed that data from financial reports and literature (i.e. not peer reviewed) were true, because we could not control these data. Per definition, data taken from expert opinions are estimations, and subdivision of expenditures in terms of personnel, material and overhead was lacking. For this reason, assessing face validity was not possible. However, for the validity and to prevent double counting of spending on prevention we collected data from executive organizations, and as much as possible used published annual reports and formal balance sheets.

A third limitation of our study is the use of PARs as a methodology to allocate costs of preventive activities to disease groups. Two important limitations of the PAR methodology are that 1) some causes of disease can be synergistic so that PARs sum to more than 100%, and 2) PARs vary in the scientific literature [38].

It is difficult to judge whether our findings are generalizable to other countries due to the lack of comparable studies. Total prevention expenditures in the USA were estimated at $188 per capita in 1988 (adjusted to price level 2003) [4], which is much lower than our estimate of $832 (in PPP dollars) in 2003 ($PPP = €0.924). The proportion of health protection in total prevention expenditures was much lower (30%) than in our study (82%). In contrast, the proportions spent on health promotion and disease prevention were higher in the USA (24% and 35%, respectively) compared to our estimate (2% and 16%, respectively). However, both studies showed that the total amount spent on prevention was much higher than prevention expenditures within the NHA only. The OECD recently estimated for 22 OECD countries the expenditures on prevention and public health within the national health budget [5]. However, these estimates did not reflect the total amount spent on prevention, but rather preventive services provided in the form of public and private programs. As opposed to our study, the OECD could not estimate expenditures on prevention provided through ambulatory care, and did not include health-related expenditures (e.g. environmental health). It should be noted that our study did not aim to present a completely elaborated health account alternative for estimating national spending on prevention. It is, rather, more a complementary system using another perspective than that used for the existing statistics for prevention, and it might be an eye-opener regarding current definitions and classifications of prevention in NHA and classifications such as the SHA. Nevertheless, comprehensive testing of our approach into practice could be a step towards a better health account alternative.

The proportion of health promotion in total prevention expenditures (2%, e.g. €15 per capita) is small compared to the health burden that can be attributed to harmful health behavior. In the Netherlands 13% of the total burden of disease can be attributed to smoking, 10% to overweight and obesity, 8% to hypertension, and 4% to physical inactivity [34]. Although health promotion activities were difficult to identify in, for instance, occupational health centers and among general practitioners (GPs), this would not substantially raise a true estimate. Successful health promotion may reduce premature mortality and improve quality of life, and may save resources [39-42], but so far the evidence for this is scarce. For example, Feenstra et al. [43] showed that minimal GP counselling is an effective and cost-saving anti-smoking intervention. Dickinson et al. [44] found robust effects on hypertension for interventions aiming at improved diet, physical exercise, alcohol and sodium restriction, and fish oil supplements. Elley et al. [45] showed that verbal and written physical activity advice given by GPs with telephone follow-up is an inexpensive way of increasing activity for sedentary people, whilst enabling significant economic impact through reduction of cardiovascular (and other) morbidity and mortality. These interventions are in general not expensive. Lleras-Muney [46] showed that the benefits of education are large enough to warrant education policies being considered more seriously as a means to increase health. In health policy considerable attention is paid to the promotion of healthy behaviors [47,48], but apparently further steps are necessary to implement evidence-based and cost-effective health promotion activities on a wider scale. Research on how to improve strategies to overcome obstacles and facilitate implementation is still needed.

A significant relationship is expected between the high expenditures on prevention of infectious diseases and injuries, and the respectively low expenditures on curative services and long-term care. Strategies to combat these health problems by regulation and preventive technologies have been successful, and provide a clear motive to investigate whether the potential of preventive strategies in other public health areas (e.g. mental health, cardiovascular risk management) has been sufficiently reached [1]. Particularly mental disorders and diseases of the musculoskeletal system involve high costs of medical and social services, in sharp contrast with low expenditures on prevention. Whether or not prevention will lead to extensive savings will depend on the availability and cost effectiveness of those preventive activities. Despite some progression, preventive activities are still missing for many diseases.

Relatively high amounts are spent on health protection measures such as air quality, drinking water systems, domestic waste disposal and traffic safety measures. Some of these have provided considerable value for money. For instance, the implementation of clean water systems in American cities substantially reduced mortality in the 19^th ^and early 20^th ^century, with very favorable cost effectiveness ($500 per life year saved in 2003 dollars) [2]. This will be similar in countries with a similar developmental path. Generally, however, not much is known about the cost effectiveness of health protection measures, and available estimates range from cost saving to high amounts per life year or QALY saved. Regarding traffic safety, seat belt use is cost saving, a driver's and dual airbag cost $24,000 and $61,000 per QALY saved, respectively (in 1993 dollars) [49], whereas these estimates are generally unknown for infrastructural safety investments. Information on cost effectiveness is even more scarce for environmental safety measures, and results are generally uncertain, and more unfavorable than for many preventive measures within health care (e.g. $54,000 per life year saved for radon mitigation in houses [50]). More insight into the cost effectiveness of these preventive measures will probably save lives more effectively [6]. Given the available evidence on the relative cost effectiveness of preventive and curative interventions, it is questionable whether the current resource allocation, and particularly the low share of health promotion activities, is efficient and in line with societal preferences.

## Conclusion

We conclude that the total expenditures on health-related prevention in the Netherlands is much higher than usually reported due to the inclusion of health protection activities beyond the NHA and SHA. The low share of health promotion activities in total expenditures is noteworthy. Because the health effects of many costly health protection activities are unknown or uncertain, this can only partly be due to the lack of available evidence on effectiveness and cost effectiveness. A systematic assessment of available evidence on the cost effectiveness of preventive and curative interventions could lead to more investments in prevention directed at health behavior.

## Competing interests

The author(s) declare that they have no competing interests.

## Authors' contributions

EWB carried out the research and drafted the manuscript. WJM conceived the idea of the paper and supervised the research. All authors contributed substantially to the study design, data collection, and the manuscript. JPM acted as guarantor. All authors have read and approved the final manuscript.

## Pre-publication history

The pre-publication history for this paper can be accessed here:



## Supplementary Material

Additional file 1 An overview of prevention measures, data sources, and types of cost calculation method. Click here for file

Additional file 2 An example of allocating costs of anti-smoking medication to different disease groups. Click here for file
