# Supplementary material for: Towards a comprehensive estimate of national spending on prevention
Source: BMC Public Health. 2007 Sep 20;7:252. doi: 10.1186/1471-2458-7-252 (PMC2071917; doi:10.1186/1471-2458-7-252)
Supplement: Additional file 1 — An overview of prevention measures, data sources, and types of cost calculation method. [file 1471-2458-7-252-S1.doc]

Additional file 1: An overview of prevention measures, data sources, and types of cost calculation method.

| **Preventive measures** | **Data source** | Type of cost calculation method |
| --- | --- | --- |
| HEALTH PROMOTION | | |
| Smoking cessation campaigns/projects | Stivoro [1] | Taken directly from annual financial report |
| Educational projects at school to prevent smoking, alcohol, drugs, and gambling dependence | Expert consultation Netherlands Institute of Mental Health and Addiction (Trimbos Institute), extrapolation based on some Centers for Alcohol and Drugs dependence | Taken directly from annual financial reports and taken from expert opinion (opinion about which percentage of total costs was allocated to smoking, alcohol, drugs and gambling dependence separately) |
| Preventive measures (e.g. peer education, information, personal advisement, quick scan) to prevent alcohol, drugs, and gambling dependence | Expert consultation Netherlands Institute of Mental Health and Addiction, extrapolation based on some Centers for Alcohol and Drugs dependence, Netherlands Institute for Health Promotion and Disease Prevention [2] | Taken directly from annual financial reports and extrapolated by researchers to national level |
| Promotion of physical activity | Netherlands Institute for Sport and Physical activity [3], De Vries & Van Eck [4] | Taken directly from data source |
| Information about healthy food | Netherlands Nutrition Center [5] | Taken directly from annual financial report |
| Diet advice by Home Care Institutions | Statistics Netherlands, expert consultation Home Care Institutions | Taken directly from data source and taken from expert opinion (opinion about which percentage of total costs was allocated to prevention) |
| Youth health care | Netherlands Association of Municipal Health Services [6], expert consultation | Taken directly from data source and taken from expert opinion (opinion about which percentage of total costs was allocated to health promotion) |
| STI and Aids information and education activities | Expert consultation Rutgers Nisso Groep (Dutch expert center on sexuality), STI AIDS Netherlands [7] | Taken directly from annual financial report and taken from expert opinion (opinion about which percentage of total costs was allocated to prevention) |
| Condom use | Expert consultation SSL International | Taken from expert opinion (opinion about total expenditures on condoms in 2003) |
| Information about dental health | Expert consultation Dutch Society for Oral Health Education | Taken from expert opinion (opinion about total expenditures on information about dental health in 2003) |
| Information about traffic safety | Witte et al. [8] | Taken directly from data source |
| Prevention of home and leisure injuries | Consumer Safety Institute [9, 10] | Taken directly from annual financial report |
| Swimming education at school | Expert consultation Ministry of Education, Culture and Science | Taken from expert opinion (opinion about total expenditures on swimming education at school in 2003) |
| Prevention of sport injuries | Consumer Safety Institute [9, 10], expert consultation Netherlands Olympic Committee Netherlands Sport Confederation (NOC*NSF), expert consultation Ministry of Health, Welfare, and Sport | Taken directly from annual financial report and taken from expert opinion (opinion about total expenditures on prevention of sport injuries executed by NOC*NSF and Ministry of Health, Welfare, and Sport in 2003) |
| Sport examinations | Statistics Netherlands | Taken directly from data source |
| Information about fire safety | Consumer Safety Institute [9, 10], Netherlands Burns Foundation [11] | Taken directly from annual financial report |
| Diabetes prevention | Dutch Diabetes Research Foundation [12], expert consultation Dutch Diabetes Research Foundation | Taken directly from annual financial report and taken from expert opinion (opinion about which percentage of total costs was allocated to prevention) |
| General information about preventing cancer | Dutch Cancer Society [13] | Taken directly from annual financial report |
| Information about breast and cervix cancer screening | Netherlands Association of Municipal Health Services [6] | Taken directly from data source |
| Preventive measures mental disorders | Netherlands Association of Municipal Health Services [6], Voordouw and Schaefer [14], expert consultation National Consultancy on Prevention | Taken directly from data source and taken from expert opinion (opinion about which percentage of total costs was allocated to prevention) |
| General health promotion | Netherlands Association of Municipal Health Services [6], Netherlands Institute for Health Promotion and Disease Prevention [2] | Taken directly from annual financial report and Taken directly from data source |
| HEALTH PROTECTION | | |
| Improvement of employment conditions | Statistics Netherlands [15], Branch Organization Occupational Health Services[16] | Taken directly from data source |
| Rule enforcement of employment conditions | Labor Inspectorate [17] | Taken directly from annual financial report |
| Rule enforcement of drugs, alcohol, and smoking bans | Expert consultation Food and Consumer Product Safety Authority | Taken directly from data source and taken from expert opinion (opinion about which percentage of total costs was allocated to smoking, alcohol, and drugs separately) |
| Reduction of noise pollution | Statistics Netherlands, Netherlands Environmental Assessment Agency [18], expert consultation Netherlands Environmental Assessment Agency | Taken directly from data source and taken from expert opinion (more details of intervention types and their costs were given; e.g. total costs of noise barriers in 2003) |
| Reduction discharge waste materials | Statistics Netherlands, Netherlands Environmental Assessment Agency [18], expert consultation Netherlands Environmental Assessment Agency | Taken directly from data source and taken from expert opinion (more details of intervention types and their costs were given; e.g. total costs of domestic rubbish in 2003) |
| Activities to guarantee fire, product, traffic and environmental safety, and to prevent violence | Statistics Netherlands, extrapolation based on some fire departments, expert consultation of Ministry of Interior and Kingdom Relations, Langeveld & Schoon [19], Wesemann [20], Netherlands Association of Municipal Health Services [6], expert consultation of Ministry of Justice, expert consultation Inspectorate of Ministry of Housing, Spatial Planning and the Environment, expert consultation Food and Consumer Product Safety Authority | Taken directly from data source and taken from expert opinion (opinion about which percentage of total costs was allocated to prevention). In case of total cost calculation of activities to guarantee fire safety researchers extrapolated costs to national level. |
| Rule enforcement of house quality | Statistics Netherlands, Netherlands Environmental Assessment Agency [18], expert consultation Netherlands Environmental Assessment Agency, expert consultation Inspectorate of Ministry of Housing, Spatial Planning and the Environment | Taken directly from data source and taken from expert opinion (opinion about total expenditures on house quality and which percentage of total costs was allocated to prevention in 2003) |
| Activities concerning food safety, quality of drinking and swimming water, and domestic waste disposal | Dutch Waste Consultation Body [21], Bronda [22], expert consultation Inspectorate of Ministry of Housing, Spatial Planning and the Environment, expert consultation Food and Consumer Product Safety Authority, Association of Dutch Water Companies [23] | Taken directly from annual financial report, Taken directly from data source and taken from expert opinion (opinion about which percentage of total costs of food safety was allocated to prevention in 2003) |
| Inspections of hygiene in sauna, day-care centers, and sex houses | Netherlands Association of Municipal Health Services [6] | Taken directly from data source |
| DISEASE PREVENTION | | |
| Vaccination | | |
| National Vaccination Program | Expert consultation Central Agency for Health Care Tariffs | Taken directly from data source and taken from expert opinion (opinion about total costs of National Vaccination Program in 2003) |
| Hepatitis B vaccination | National Institute for Public Health and the Environment [24] | Taken directly from data source |
| Flu vaccination | Ministry of Health, Welfare, and Sport [25] | Taken directly from data source |
| Tropical disease vaccination | Netherlands Association of Municipal Health Services [6] | Taken directly from data source |
| Screening | | |
| TBC screening | Rutz et al. [26], Netherlands Association of Municipal Health Services [6] | Taken directly from data source |
| Screening of sexually transmitted diseases | Netherlands Association of Municipal Health Services [6] | Taken directly from data source |
| Screening of hepatitis B | National Institute for Public Health and the Environment [24] | Taken directly from data source |
| Screening of congenital hypercholesterolemy | Witte et al. [8] | Taken directly from data source |
| Preventive dental health | Expert consultation National Institute for Public Health and the Environment | Taken directly from data source and taken from expert opinion (opinion about total expenditures on screening dental health in 2003) |
| Pre and postnatal screening | Health Care Insurance Board [27] | Taken directly from data source |
| Heel prick (phenylketonuria test) | Expert consultation Central Agency for Health Care Tariffs, expert consultation Dutch Foundation of Graft Administration | Taken from expert opinion (opinion about total cost of heel prick in 2003) |
| Accompaniment of pregnancy | Statistics Netherlands, Anthony et al. [28], expert consultation Central Agency for Health Care Tariffs | Taken directly from data source and taken from expert opinion (opinion about the average cost per patient of pregnancy accompaniment) |
| Youth health care | Netherlands Association of Municipal Health Services [6], expert consultation | Taken directly from data source and taken from expert opinion (opinion about which percentage of total costs was allocated to screening) |
| Screening for breast cancer | Witte et al. [8] | Taken directly from data source |
| Screening for cervix cancer | Witte et al. [8] | Taken directly from data source |
| Medical examinations | Statistics Netherlands [15] | Taken directly from data source |
| Preventive medication | | |
| Anti-smoking medication | Dutch Foundation for Pharmaceutical Statistics | Taken directly from data source |
| Cholesterol suppressants | Witte et al. [8] | Taken directly from data source |
| Blood pressure suppressants | Witte et al. [8] | Taken directly from data source |
| Anti-contraceptives | Dutch Foundation for Pharmaceutical Statistics | Taken directly from data source |
| Anti-osteoporosis medication | Taken directly from data source | Taken directly from data source |
| Vitamins | Dutch Foundation for Pharmaceutical Statistics | Taken directly from data source |

References Additional file 1
