# Supplementary material for: Towards a comprehensive estimate of national spending on prevention
Source: BMC Public Health. 2007 Sep 20;7:252. doi: 10.1186/1471-2458-7-252 (PMC2071917; doi:10.1186/1471-2458-7-252)
Supplement: Additional file 2 — An example of allocating costs of anti-smoking medication to different disease groups. [file 1471-2458-7-252-S2.doc]

Additional file 2: An example of allocating costs of anti-smoking medication to different disease groups.
